# Geographical distribution of potential mechanical vectors implicated in Surra transmission in Spain: an entomological perspective

**DOI:** 10.1186/s13071-025-06922-9

**Published:** 2025-07-28

**Authors:** Adrián Melián-Henríquez, María Teresa Tejedor-Junco, Daniel Bravo-Barriga, Mikel Alexander González, Pedro María Alarcón-Elbal, Carlos Barceló, Ignacio Ruiz-Arrondo, Massimo Paone, Giuliano Cecchi, Juan Alberto Corbera

**Affiliations:** 1 Research Institute of Biomedical and Health Sciences, University of Las Palmas de Gran Canaria, Canary Islands, Spain; 2 Department of Animal Health (Parasitology and Parasitic Diseases), Faculty of Veterinary Medicine, University of Cordoba, Cordoba, Spain; 3 Departamento de Biología de la Conservación y Cambio Global, Estación Biológica de Doñana (EBD-CSIC), Seville, Spain; 4 Departamento de Soluciones Técnicas y Entomología, Grupo SASTI, Villaverde, Madrid, Spain; 5 Department of Animal Production and Health, Faculty of Veterinary Medicine, Public Veterinary Health and Food Science and Technology, Universidad Cardenal Herrera-CEU, Valencia, Spain; 6 Applied Zoology and Animal Conservation Group, Faculty of Sciences, University of the Balearic Islands, Palma, Spain; 7 Department of Animal Pathology. Faculty of Veterinary Sciences, Instituto Agroalimentario de Aragón-IA2 (Universidad de Zaragoza-CITA), Zaragoza, Spain; 8 Animal Production and Health Division, Food and Agriculture Organization of the United Nations (FAO), 00153 Rome, Italy; 9 Faculty of Veterinary, Hospital Clínico Veterinario, University of Las Palmas de Gran Canaria, Canary Islands, Spain

**Keywords:** Diptera, Surra, Maps, Muscidae, Hippoboscidae, Tabanidae, Citizen science databases, Atlas

## Abstract

**Background:**

Haematophagous Diptera can transmit a wide range of diseases to both humans and animals. Some species of the *Trypanosoma* genus rely on these vectors for transmission, either cyclically or mechanically. *Trypanosoma evansi*, the causative agent of Surra, is the only African-origin trypanosome species detected in Spain to date, which is mechanically transmitted.

**Methods:**

To assess the occurrence and distribution of potential mechanical vectors at the national level, a systematic review was conducted on the Hippoboscidae, Muscidae and Tabanidae families. The review followed the methodology established by the Food and Agriculture Organization of the United Nations (FAO) and adhered to PRISMA guidelines. Data were compiled from 43 peer-reviewed scientific publications and four citizen science digital databases.

**Results:**

The review identified three genera belonging to the Hippoboscidae, two of the Muscidae and ten of the Tabanidae families. Genus-level distribution maps were generated for each group.

**Conclusions:**

This atlas serves as a valuable tool for the prevention and control of vector-borne animal trypanosomosis in Spain. Nonetheless, further studies on the distribution, ecology and behaviour of haematophagous dipterans are essential to better understand their role in disease transmission and their potential impact on future outbreaks.

**Graphical abstract:**

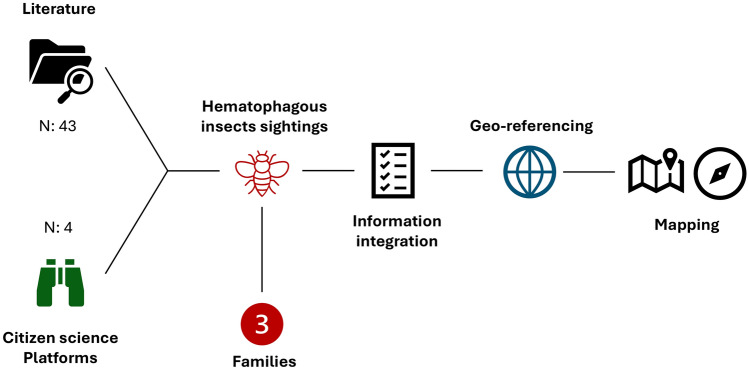

**Supplementary Information:**

The online version contains supplementary material available at 10.1186/s13071-025-06922-9.

## Background

Haematophagous arthropods play a critical role in both human and animal health [69]. Their blood-feeding behaviour can cause direct harm, including irritation, immune reactions and localised trauma. Indirectly, they act as vectors for a wide range of infectious agents, facilitating both mechanical and biological (i.e. cyclical) transmission [87]. Among these pathogens, protozoans of the genus *Trypanosoma* (Gruby, 1843; Kinetoplastida: Trypanosomatidae) are of particular concern owing to their impact on veterinary and human medicine.

Animal trypanosomosis, transmitted by vectors and manifesting as either Nagana and Surra, poses a significant barrier to livestock production across many African regions. Nagana is caused by *Trypanosoma brucei* (Plimmer & Bradford, 1899), *Trypanosoma vivax* (Ziemann, 1905) and *Trypanosoma congolense* (Broden, 1904), while *Trypanosoma evansi* (Chauvrat, 1896) causes Surra [17]. Unlike other trypanosomes, *T. evansi* does not need a biological vector and is instead transmitted mechanically by haematophagous insects [18, 72]. However, the parasite’s limited survival time within the insect’s mouthparts constrains transmission efficiency.

Haematophagous dipterans are the most relevant vectors of *T. evansi*, owing to their high mobility and frequent feeding patterns [18, 67, 87]. Several studies highlighted the role of different insect taxa in the transmission of Surra to mammals, including biting flies of the tribe Stomoxyini (Diptera: Muscidae) (*Stomoxys* spp. and *Haematobia* spp.), tabanids (Diptera: Tabanidae) (*Tabanus* spp., *Atylotus* spp. and *Chrysops* spp., among others) and louse flies (Diptera: Hippoboscidae) (*Hippobosca* spp., *Lipoptena* spp. and *Melophagus* spp.) [20].

Surra is mainly found in North Africa, Northeast Africa, Latin America, the Middle East and Asia. However, outbreaks have also been reported in Europe, including France (Aveyron Department) and Spain. In the latter country, cases were recorded in the province of Alicante (mainland Spain), linked to the movement of infected animals from the Canary Islands [15, 82]. The first confirmed case of Surra in Spain occurred in 1997 on the Island of Gran Canaria [46], which led to subsequent studies to assess the parasite’s prevalence in dromedaries and other livestock, as well as the potential role of rodents and haematophagous insects in disease maintenance [60, 73–75].

Although no new seropositive cases of Surra have been reported in the Canary Islands since 2022 [57, 83], ongoing surveillance and research into disease dynamics remain essential to prevent future outbreaks. This is particularly relevant, as Spain may act as an entry point for the disease into mainland Europe. In this context, the current study aims to compile a comprehensive atlas of the distribution of potential *T. evansi* vectors across Spain, within the framework of the COMBAT project (COntrolling and progressively Minimizing the Burden of Animal Trypanosomosis) [8]. This work complements the recently published national atlas of Surra in the country [57].

## Methods

### Search protocol and selection criteria

A systematic review was carried out in accordance with the Preferred Reporting Items for Systematic reviews and Meta-Analyses (PRISMA) guidelines [59], and following the methodology established by the Food and Agriculture Organization of the United Nations (FAO) for continental and national atlases of *Glossina* spp. and African trypanosomes [1, 11–14, 21, 42, 65, 71, 80]. In addition, scientific repositories and search engines, including PubMed^®^, Scopus, Google Scholar and ResearchGate were used, without date restrictions. The collection and analysis period of the data from these platforms extended from February to December 2024.

The review focused on insect taxa identified as potential mechanical vectors of *T. evansi*, with particular attention on the families Muscidae, Hippoboscidae and Tabanidae [15, 17–19, 56, 75, 82]. Genera within each family were selected on the basis of prior literature*,* including *Stomoxys* and *Haematobia* for Muscidae [17, 18], and mammophilic genera for Hippoboscidae (*Hippobosca*, *Lipoptena,* and *Melophagus*) [84]. For the Tabanidae, the classification followed the nomenclature proposed by Portillo (2002) for horsefly in Spain, encompassing *Atylotus*, *Chrysops*, *Dasyrhamphis*, *Haematopota*, *Hybomitra*, *Nemorius*, *Pangonius*, *Philipomyia*, *Silvius*, and *Tabanus*.

Keyword searches were conducted in Spanish, French and English, using terms such as ‘Diptera’, ‘Spain’, ‘Canary Islands’, ‘Balearic Islands’, ‘mainland Spain’, ‘Haematophagous’, ‘Muscidae’, ‘Hippoboscidae’, ‘Tabanidae’, ‘*Stomoxyini*’, ‘*Stomoxys*’, ‘*Haematobia*’, ‘*Lipoptena*’, ‘*Hippobosca*’, ‘*Melophagus*’, ‘*Tabanus*’, ‘*Haematopota*’, ‘*Dasyrhamphis*”, ‘*Atylotus*”, ‘*Pangonius*’, ‘*Silvius*’, ‘*Chrysops*’, ‘*Hybomitra*’, ‘*Nemorius*’, ‘*Philipomyia*’, ‘*Trypanosoma evansi*’ and ‘Surra’. Approximately less than 10% of the studies identified were excluded owing to access restrictions, often related to publication age or paywalls. However, some were retained indirectly through citations in other included sources.

### Citizen science platforms

Data from citizen science platforms were obtained from four sources: iNaturalist (Nugent, 2018), GBIF.org, Biodiversidad Virtual (https://www.biodiversidadvirtual.org) and the Biodiversity Data Bank of the Canary Islands (BDBC) (https://www.biodiversidadcanarias.es/biota). Searches were conducted using insect genera or species keywords, and results were filtered by country (last accessed: 11 March 2025). These platforms primarily provide presence-only records, often accompanied by precise geographical coordinates, occurrence dates and photographic evidence.

Only records validated by experts were retained. When expert validation was unavailable, all submitted images were manually reviewed. Duplicate entries across platforms were cross-checked and excluded.

### Digital repository and database

The literature retrieved through the systematic review was compiled in a digital repository, and the following information were extracted regarding the occurrence of target insect taxa:

- **Sources:** Full reference details of each document, including authors, title, date and journal.
- **Site geographical data (Geo_Data_Site):** Sampling locations reported in the documents, georeferenced using Google Earth or Spanish National Geographic System when not explicitly stated. Location accuracy varied, from specific coordinates to approximate central points of municipalities. For broad designations (e.g., nature reserves), the centroid of the defined area was used.
- **Site entomological data (Entomo_Data_Site):** Descriptions of insect findings at each site, including collection date, taxonomic identification (family, genus, species, subspecies), number of individuals, trap types and survey duration.
- **Trap geographical data (Geo_Data_Trap):** Exact coordinates of sampling sites when such detail was available in the source publications.
- **Trap entomological data (Entomo_Data_Trap):** Entomological data associated with specific trap sites, mirroring the structure of *Entomo_Data_Site* but with finer spatial resolution.

### Mapping

Genus-level occurrence maps for each insect family were generated using QGIS Geographic Information System (version 3.34.11, QGIS Association). The data presented on these maps were separated into two categories: literature-based records and citizen science records. To harmonise spatial reporting, particularly for entries derived from broader administrative units, the number of genera recorded per province was also indicated on the maps.

## Results

This study analysed 105 species across 15 genera and three families, on the basis of a total of 2709 data records published between 1951 and 2024 throughout Spain. From the literature, 43 sources were consulted (40 scientific publications, two books and one doctoral thesis) (see Additional file 1: text S1 for more information), yielding 1088 presence records. From citizen science platforms, 1621 presence records were included. Although GBIF.org aggregates records from iNaturalist and Biodiversidad Virtual, 65% originated from Biodiversidad Virtual, 20% from iNaturalist and 15% from GBIF. Biodiversity Data Bank of the Canary Islands (BDBC) records were limited to the Canary Islands and lacked specific coordinates.

A total of 323 locations were georeferenced from the literature: 23 at the trap level or exact capture site, and 300 at broader spatial resolutions. In contrast, 90% of citizen science records were georeferenced with precise coordinates.

Some literature sources included quantitative trapping data. The total number of captured or observed insects during the study period was 20,076 individuals. Of the 1088 literature-based records, 18,455 insects were quantified: 86.5% belonged to the Muscidae family, 13.3% to the Tabanidae family and 0.2% to the Hippoboscidae. Each citizen science record was assumed to represent a single observed individual, totalling 1621 insects, of which 64.8% were Tabanidae, 19.2% Muscidae and 16% Hippoboscidae.

Table 1 summarises the families and genera included in the study, indicating the number of species per genus, number of provinces in which each genus was recorded, host species and trapping methods cited in the literature. Species-level details can be found in Additional file 2: Table S1.

**Table 1:** Summary of the blood-feeding dipteran families analysed in Spain (from 1951 to 2024). *N*: number; ND: not described

Family	Genus	Species (*N*)	Provinces (*N*)	Cited hosts	Cited capture methods
Hippoboscidae	*Hippobosca* Linnaeus, 1758	2	44	Equids, bovids, canids, felids, human	Trap (Sticky), sweep
	*Lipoptena* Nitzsch, 1818	3	16	Cervids, human	Trap (Suction)
	*Melophagus* Latreille, 1802	1	6	*Ovis aries*, cervids	ND
Muscidae	*Stomoxys* Geoffroy, 1762	1	44	Equids, ruminants, camelids, human	Trap (Sticky, suction, BG-sentinel, Nzi), sweep
	*Haematobia* Lepeletier & Serville, 1828	2	14	Bovids, human	Trap (Sticky, suction, CDC-Miniature light trap)
Tabanidae	*Atylotus* Osten-Sacken, 1876	9	20	Equids, bovids	Trap (Canopy, Malaise), sweep
	*Chrysops* Meigen, 1803	7	40	Equids, bovids, cervids	Trap (Canopy, Malaise), sweep
	*Dasyrhamphis* Enderlein, 1922	3	25	Equids	Sweep
	*Haematopota* Meigen, 1803	12	38	Equids, bovids	Trap (Canopy, Malaise), sweep
	*Hybomitra* Enderlein, 1922	12	23	Equids, bovids	Trap (Malaise, H-trap), sweep
	*Nemorius* Rondani, 1856	1	3	Equids, bovids	ND
	*Pangonius* Latreille, 1802	14	30	Equids, human	Sweep
	*Philipomyia* Olsufjev, 1964	2	21	Equids, bovids	Trap (Canopy, Malaise), sweep
	*Silvius* Meigen, 1820	3	8	Equids, bovids	Trap (Malaise)
	*Tabanus* Linnaeus, 1758	33	44	Equids, bovids, human	Trap (Canopy, Malaise, Sticky, H-trap), sweep

### Hippoboscidae family

**Fig. 1:**
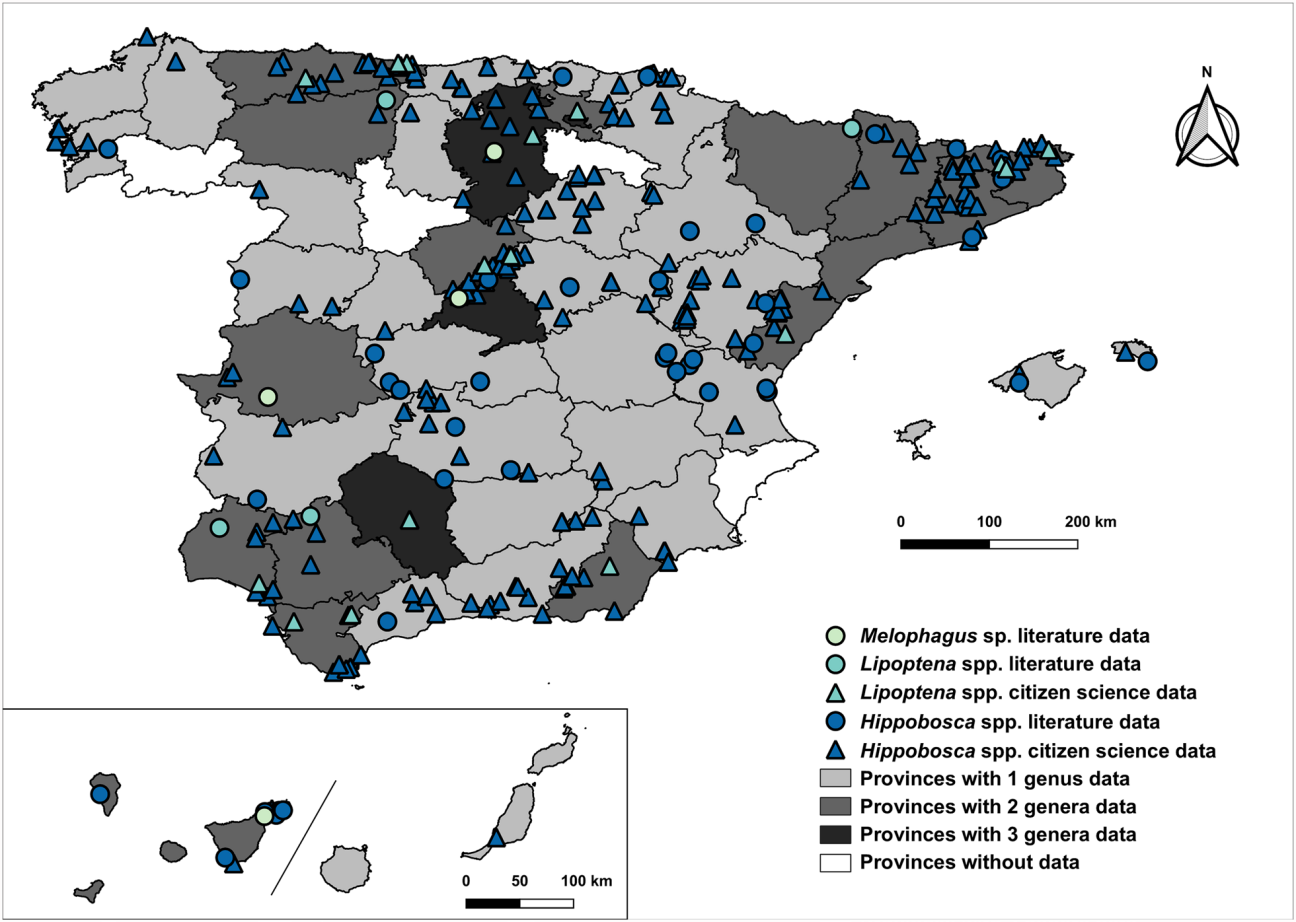
Geographic distribution of Hippoboscidae family in Spain (1951–2024). The map includes mainland Spain (centre), the Balearic Islands (right) and the Canary Islands (bottom left)

Three mammophilic genera were reviewed within the Hippoboscidae family (Fig. 1). The *Hippobosca* genus showed the highest number of observations, primarily in northern, north-eastern, central and central-eastern and southern mainland Spain, with additional sightings in both Canary Islands provinces, and in the Balearic Islands.

The *Lipoptena* genus was mostly observed in the northern, north-eastern and south-western mainland Spain, with no records from the Canary or the Balearic islands.

Finally, the *Melophagus* genus was represented by a few isolated in northern, central and central-western mainland Spain, as well as the western Canary Islands.

### Muscidae family

**Fig. 2:**
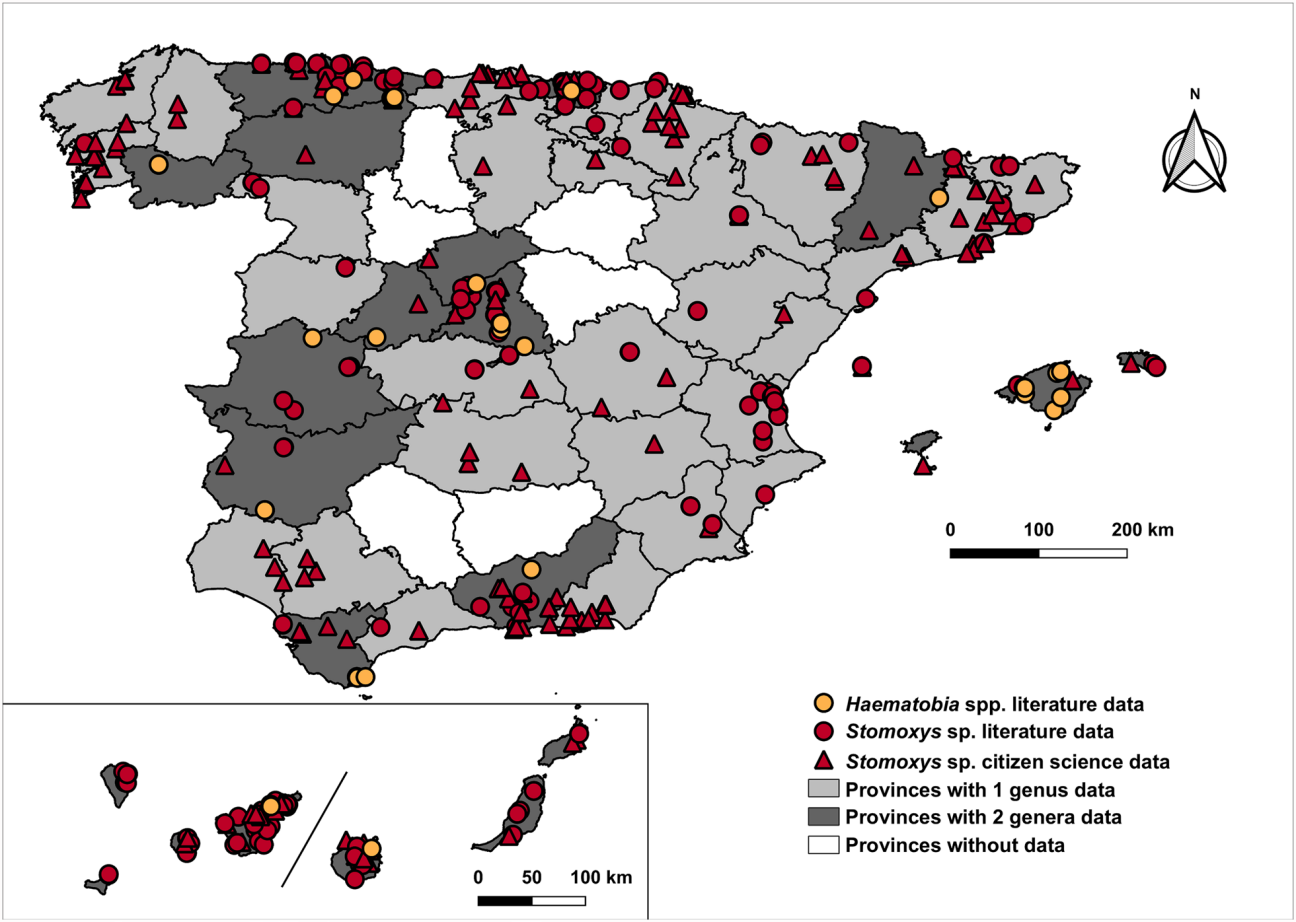
Geographic distribution of Muscidae family in Spain (1951–2024). The map shows mainland Spain (centre), the Balearic Islands (right) and the Canary Islands (bottom left)

Two genera were reviewed within the Muscidae family (Fig. 2). Sightings of the *Stomoxys* genus were distributed across most of Spain, especially in the northern, southern and central regions of mainland regions. In addition, all islands of the Balearic and Canary archipelagos reported sightings. Observations of the genus *Haematobia* were concentrated in the northern, central and southern mainland, as well as the Balearic Islands, with a smaller number of reports from the Canary Islands.

### Tabanidae family

**Fig. 3:**
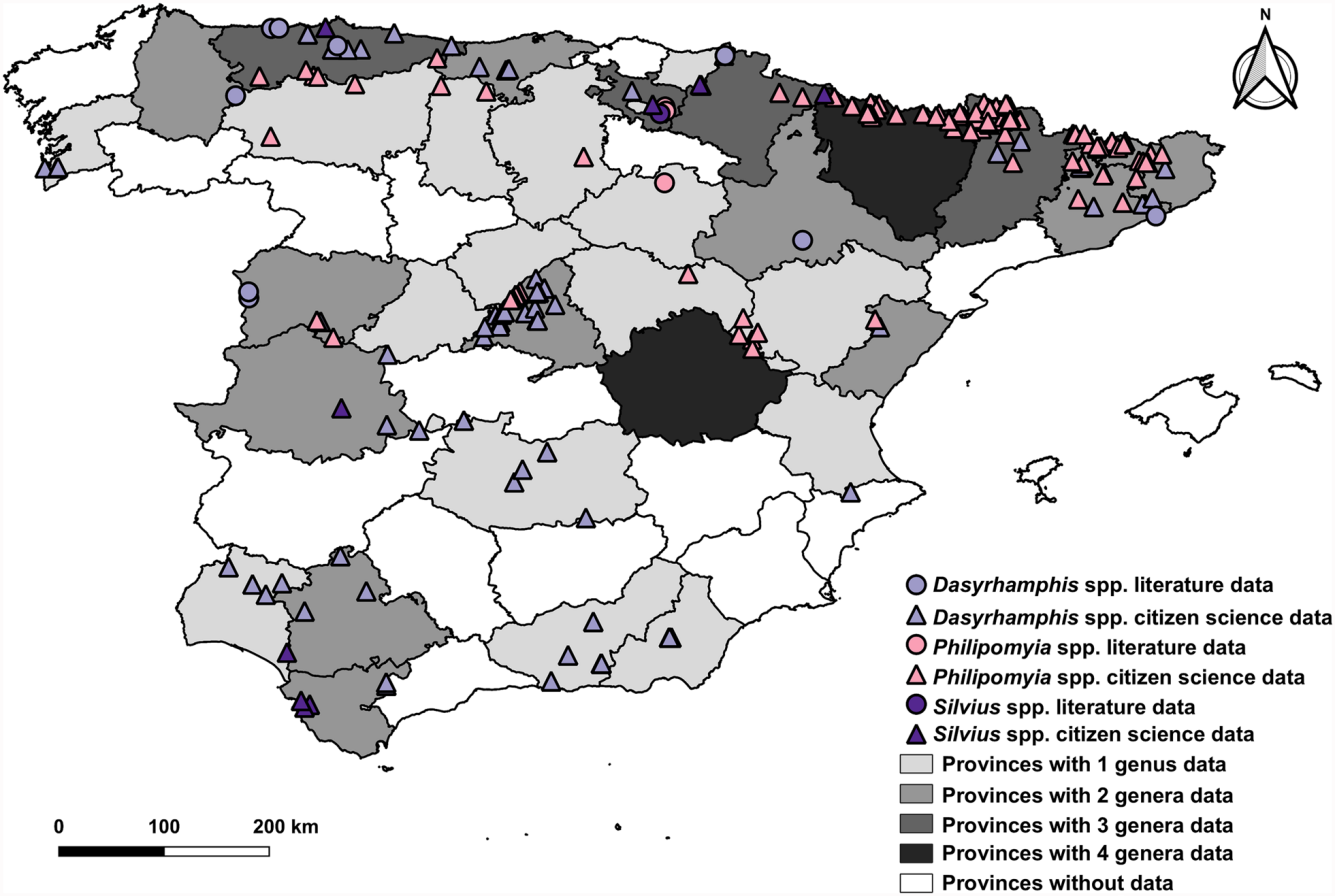
Geographic distribution of the genera *Dasyrhamphis*, *Philipomyia*, *Silvius* and *Nemorius* in Spain (1951–2024). The map includes mainland Spain (centre) and the Balearic Islands (right)

A total of ten genera within the Tabanidae family were studied. Figure 3 shows the occurrence for four of the genera: *Dasyrhamphis*, *Philipomyia*, *Silvius* and *Nemorius*. The *Dasyrhamphis* genus was found mainly in central, southern and north-western mainland Spain, whereas the *Philipomyia* genus was predominantly observed in north-eastern mainland Spain. The *Silvius* genus was limited to a few sightings in northern and southern mainland Spain. Finally, no specific coordinates were available for *Nemorius* genus; however, its presence was reported in central and northern mainland Spain.

**Fig. 4:**
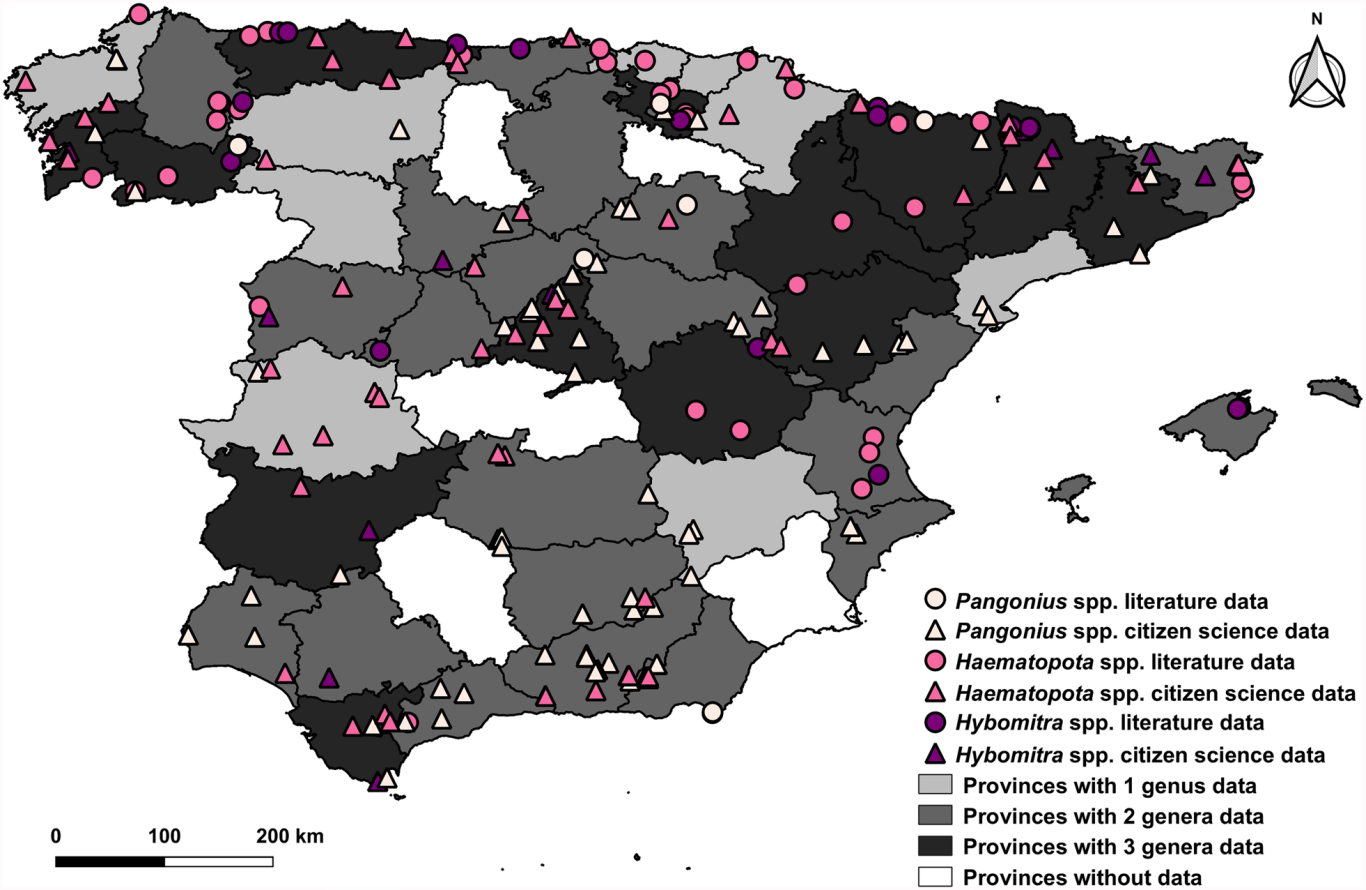
Geographic distribution of the genera *Pangonius*, *Haematopota* and *Hybomitra* in Spain (1951–2024). The map includes mainland Spain (centre) and the Balearic Islands (right)

The sightings of the *Pangonius*, *Haematopota* and *Hybomitra* genera are shown in Fig. 4. For *Pangonius*, sightings are distributed in the central, southern and north-eastern regions of mainland Spain. For *Haematopota* and *Hybomitra*, sightings were recorded both in mainland Spain (especially in the north and centre) and in the Balearic Islands.

**Fig. 5:**
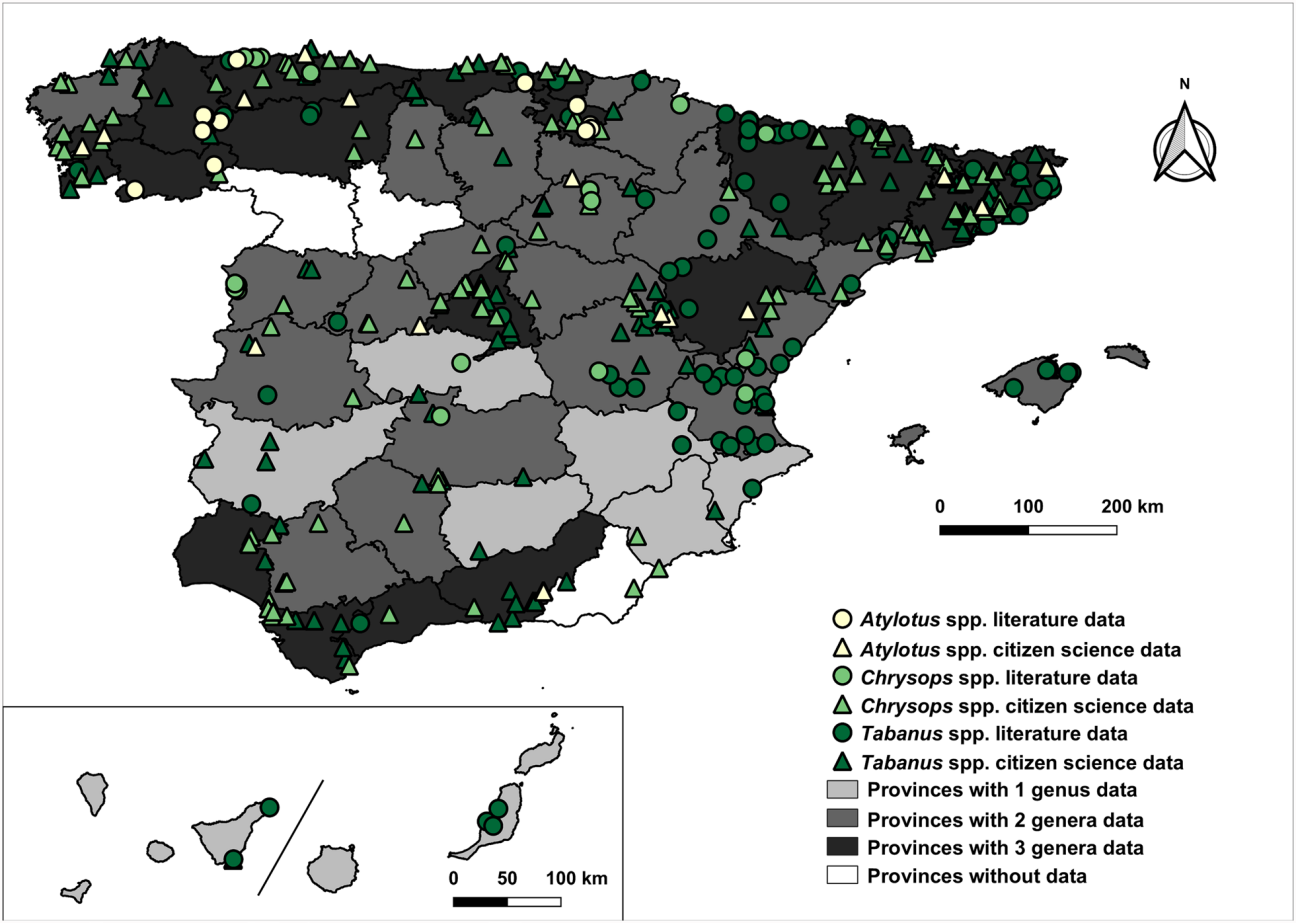
Geographic distribution of the genera *Atylotus*, *Chrysops* and *Tabanus* in Spain (1951–2024). The map includes mainland Spain (centre), the Balearic Islands (right) and the Canary Islands (bottom left)

Finally, Fig. 5 shows records for the *Atylotus*, *Chrysops* and *Tabanus* genera. The genus *Atylotus* was mostly observed in northern mainland Spain, though unspecified records exist for the Balearic Islands. The genus *Chrysops* was mostly restricted to central and northern mainland Spain. The genus *Tabanus* was reported throughout Spain, including the Canary and Balearic Islands, with notable concentrations in the north-east and central mainland.

## Discussion

This study, developed within the framework of the COMBAT project [8], represents the first comprehensive compilation of information on the potential vectors of Surra in Spain. Previous national atlases on trypanosome control and its vectors in African countries typically included both a tsetse component and an animal infection component [1, 21, 42, 65, 71]; however, this study focused exclusively on the entomological component, specifically on the three different dipteran families that can potentially transmit Surra in Spain. The Surra atlas for Spain has already been published [57], and to date, no vectors belonging to the *Glossina* genus have been reported in the country.

A notable gap in the literature relating vector presence to Surra in Spain became evident during the compilation of information sources. The present data reveal a high diversity of horsefly genera in the country, particularly in mainland Spain. Among these, the *Tabanus* genus stands out for its species richness and high abundance, and is the only genus found across mainland Spain, the Balearic Islands and the Canary Islands. Likewise, the *Hippobosca* genus (especially *Hippobosca equina* Linnaeus, 1758), *Melophagus* genus (*Melophagus ovinus* (Linnaeus, 1758)) and *Stomoxys* genus (*Stomoxys calcitrans* (Linnaeus, 1758)) are generally distributed throughout Spain. Among these, *S. calcitrans* is particularly abundant and found in diverse biotopes and environmental conditions. However, while the literature describes the distribution of *M. ovinus* as widespread, there is a notable lack of georeferenced sightings. It is also worth noting that only one record exists for the genus *Haematobia* (*Haematobia titilans* (Bezzi, 1907)) in the Canary Islands, with no subsequent observations to date.

Given the wide distribution of all the genera recorded in this study, potential re-emergent Surra outbreaks in Spain would likely encounter an abundance of competent mechanical vectors, as seen in past episodes in the Canary Islands and Alicante [82, 83]. In such scenarios, Surra could not only spread across Spain, but also pose a threat to continental Europe, as demonstrated by the 2008 outbreak in Aveyron (France), linked to the transport of infected dromedaries from the Canary Islands [15].

In addition to their potential role Surra transmission, bites from certain haematophagous flies pose an occupational hazard and a considerable nuisance in rural areas of Europe [47, 54]. The study of these brachycerans flies in Spain has been far less extensive than that of nematoceran vectors, such as mosquitoes, sandflies and biting midges [9, 29] owing to their limited public health impact in urban settings and the lack of standardised trapping methods [77]. Another key factor is that these brachycerans rely on less refined capture methods than nematocerans do [48, 78, 85]. This methodology gap warrants further exploration to enhance our understanding of these vectors [2, 58]. For example, it would be necessary to standardise and optimise trapping methods for each of the families, especially in the case of louse flies, which generally require direct host contact for capture. In this context, carbon dioxide traps have recently proven effective for monitoring *Lipoptena* species in Spain [44]. For tabanids, comparative studies are underway to evaluate existing trap types and explore modifications for enhanced performance [48], also serving as mechanical control strategies. In North America, horseflies are often sampled using large open-style traps equipped with shiny black spherical targets, such as Malaise, Canopy, Box, Greenhead, Manitoba and Epps traps [7]. In Spain, tabanids were successfully collected using custom-made Canopy traps, sweep nets and Malaise traps [43].

Notably, the genera included in this atlas do not reflect the full diversity of haematophagous flies in Spain. Furthermore, the absence of data from certain regions does not necessarily imply species absence. For example, within other blood-feeding Hippoboscidae within the Ornithomyinae subfamily, primarily parasitised birds, are not included here owing to their limited relevance for Surra transmission.

This review highlights that some genera could act as potential vectors not only for Surra, but for a broad range of other diseases. For instance, the *Stomoxys* genus, and other muscids have been implicated in the transmission of salmonellosis, shigellosis, bacillary dysentery and even aspergillosis [5]. *Stomoxys* species are considered potential vectors of anthrax, a role they share with horseflies such as *Chrysops* spp. and *Tabanus* spp., both of which have been associated with tularemia transmission [6, 86]. Other studies highlight the role of the *Stomoxys* in the mechanical transmission of viruses (e.g., equine infectious anaemia virus, African swine fever virus, West Nile virus or Rift Valley virus), protozoa (*Besnoitia besnoiti*; Besnoit and Robin, 1912) and helminths (*Habronema microstoma*; Schneider, 1866) [5], facilitated by their frequent and persistent feeding behaviour [22]. Similarly, Hippoboscidae (*Hippobosca*, *Melophagus* and *Lipoptena*), have been linked to viruses (e.g., border disease virus or bluetongue virus) and bacteria (*Rickettsia* spp., *Borrelia* spp., *Bartonella* spp. or *Corynebacterium pseudotuberculosis*) [23].

The distribution maps presented in this study are subject to several limitations. They are based on local studies and citizen science data, with heterogeneous sampling efforts and methodologies. This reliance introduces potential biases and may not reflect current distributions or ecological dynamics. In addition, while citizen science data are valuable, they are not always consistent in terms of quality and geographic coverage. These issues underscore the urgent need for standardised, large-scale field surveys to produce more robust and updated distribution data. New surveys at the meso- and microscales could provide crucial insights into species ecology and interactions with potential hosts. Citizen science platforms offer significant potential for this purpose, especially those with strong expert participation and photographic validation, as demonstrated by apps already implemented in Spain [45, 62]. Advances in artificial intelligence for insects recognition also offer exciting prospects for integrating automated data processing, whether by scientists or citizens [26, 50, 70]. Ultimately, cooperation between researchers, citizens, public health authorities and stakeholders could foster a more holistic and integrated vector surveillance and control programs in Spain and beyond.

## Conclusions

This work represents a valuable contribution to the understanding of the epidemiology of *T. evansi* in Spain, as it is the first to consolidate and integrate information on the presence of its potential dipteran vectors across the country. Documenting the occurrence of these vector enables health authorities to implement more effective control and prevention strategies, as well as to respond promptly in the event of new outbreaks. Despite the findings presented, additional studies are needed to further characterise the distribution of these vectors throughout the territory. In addition, the integration of citizen science platforms as a complementary tool for scientific research, significantly enhancing our understanding and surveillance of vector populations in Spain.

## Supplementary Information

Additional file 1.

Additional file 2.

## Data Availability

Data supporting the main conclusions of this study are included in the manuscript.

## Abbreviations

COMBAT: COntrolling and progressively minimizing the burden of animal trypanosomosis
FAO: Food and Agriculture Organization of the United Nations
PRISMA: Preferred Reporting Items for Systematic reviews and Meta-Analyses
