# Supplementary material for: Geographical distribution of potential mechanical vectors implicated in Surra transmission in Spain: an entomological perspective
Source: Parasit Vectors. 2025 Jul 28;18:305. doi: 10.1186/s13071-025-06922-9 (PMC12305963; doi:10.1186/s13071-025-06922-9)
Supplement: Supplementary file 1 — Additional file 1. [file 13071_2025_6922_MOESM1_ESM.docx]

**Supplementary file 2**

Scientific papers used as sources to develop the atlas

1. Báez M. Los hipobóscidos de las Islas Canarias (Diptera, Hippoboscidae). Boletín de la Estación Central de Ecología. 1978. 7; 13:59-72.
2. Báez M., Portillo M. Los tabánidos de las Islas Canarias (Dip. Tabanidae). Boletín de la Asociación española de Entomología. 1982. 5:87-91.
3. Báez M. Dípteros de Canarias VII: Muscinae (Dipt., Muscidae). Vieraea. 1982. 11; 1-2:171-192.
4. Cabanillas D. Ampliación de la distribución conocida de coleópteros, dípteros e himenópteros sarcosaprófagos (Arthropoda: Insecta) con potencial interés forense en la península ibérica. Graellsia. 2022 Apr 28;78. 1:155–5.
5. ‌Campillo M., Castañón L., Reguera A. Índice – Catálogo de zooparásitos ibéricos. Universidad de León. 1994. ISBN: 84-7719-403-3.
6. Compte A. Los tabánidos de Mallorca. Bolletí de la Societat D'història Natural de Les Balears. 1958. 4:13-22.
7. Domínguez G. North Spain (Burgos) wild mammals ectoparasites. Parasite. 2004 Sep;11. 3:267–72.
8. ‌García A. Eficacia de la cipermetrina (HIGH-CIS) en el control de ectoparásitos (Moscas y garrapatas) en rumiantes: ensayos de campo. Servicio Central de Publicaciones del Gobierno Vasco. 1993. ISBN: 84-457-0261-0.
9. García C., Valcárcel F., Corchero J., Olmeda A., Pérez-Jiménez J. Contribución al estudio de las parasitosis del ciervo (*Cervus elaphus*) en las provincias de Toledo y Ciudad Real (Castilla-La Mancha, España). Ecología. 2000. 14: 235-249.
10. González MA., Stokes JE., Bravo-Barriga D. Diversity and abundance of tabanids in Northern Spain. Parasitology Research. 2021 Nov 24;121. 1:87–96.
11. ‌González MA., Bravo-Barriga D., Barrio E., Frontera E., Ruiz-Arrondo I. Severe Skin Lesions Caused by Persistent Bites of the Stable Fly *Stomoxys calcitrans* (Diptera: Muscidae) in a Donkey Sanctuary of Western Spain. Journal of Equine Veterinary Science. 2022 Sep 1; 116:104056–6.
12. ‌González MA., Ruiz-Arrondo I., Bravo-Barriga D., Cervera-Acedo C., Santibáñez P., Oteo JA., Mirada M., Barceló C. Surveillance and screening of Stomoxyinae flies from Mallorca Island (Spain) reveal the absence of selected pathogens but confirm the presence of the endosymbiotic bacterium Wolbachia pipientis. Research in Veterinary Science. 2024 May; 171:105206.
13. ‌González MA., Mirada M., Barceló C. Nuevas aportaciones de tábanos (Diptera, Tabanidae) para la fauna balear. Boletín de la Asociación española de Entomología. 2024. 48. 1-2: 59-67.
14. González MA., Ruiz-Arrondo I., Magallanes S., Oboňa J., Ruiz-López MJ., Figuerola J. Molecular and morphological analysis revealed a new Lipoptena species (Diptera: Hippoboscidae) in southern Spain harbouring *Coxiella burnetii* and bacterial endosymbionts. Veterinary Parasitology. 2024 Sep 3; 332:110300–0.
15. ‌Guzmán J., Martínez A. Contribución al estudio de *Hippobosca equina* Linnaeus, 1758 (Insecta Diptera: Hippoboscidae) en España y su interés sanitario-veterinario. Zoolentia. 2023. 3: 55-74.
16. Lacarra P., Saloña M. Nuevos datos sobre la presencia de *Stomoxys calcitrans* (Linnaeus, 1758) (Diptera, Muscidae) en explotaciones ganaderas de Vizcaya (País Vasco, Norte de España). Boletín de la Asociación española de Entomología. 2010. 34. 1-2: 199-205.
17. Leclercq M. Tabanidae (Dipt.) d’Espagne. I, *Therioplectes valenciae* N. SP. Institut royal des Sciences naturelles de Belgique. 1957 Oct. Tome XXXIII. 50.
18. Leclercq M. Tabanidae (Dipt.) d’Espagne. Bulletin des Recherches agronomiques de Gembloux. 1970. Tome V. 1-2: 281-285.
19. Leclercq M. Numerosos Tabanidae y Muscidae sobre un caballo en España y descripción de *Haematopota enriquei* NOV. SP. Graellsia. 1971. Tomo XXVI. 115-126.
20. Leclercq M. Tabanidae (Diptera) de España y Portugal. Graellsia. 1980. Tomo XXXIV. 53-57.
21. Leclercq M. Tabánidos españoles de las provincias de Oviedo y Santander. Graellsia. 1977. Tomo XXII. 219-226.
22. Martín BD., Inés M. Arthropods of forensic interest associated to pig carcasses in Aiako Harria Natural Park (Basque Country, Northern Spain). Ciencia forense: Revista aragonesa de medicina legal. 2015 Jan 1. 12: 207–28.
23. ‌Martínez A. Contributions to an understanding of the health status of the red deer (*Cervus elaphus*) from northern Spain: Parasitological studies [Ph.D. Dissertation]. Universidad de León. 2015.
24. Millán J., Ruiz-Fons F., Márquez FJ., Viota M., López-Bao JV., Martín-Mateo M. Ectoparasites of the endangered Iberian lynx *Lynx pardinus* and sympatric wild and domestic carnivores in Spain. Medical and Veterinary Entomology. 2007 Sep 26. 21. 3:248–54.
25. ‌Mora D. Datos sobre la distribución de los *Muscini* y *Stomoxyini* españoles (Diptera: Muscidae). Boletín de la Asociación española de Entomología. 1989. 13: 53-56.
26. Peris SV. Los Stomoxydinae de la Península Ibérica. Revista de la Academia de Ciencias. 1951. Tomo VI. 2: 41-49.
27. Portillo M. Tabanidae (Diptera) de España: VI. *Tabanus* Linnaeus, 1758. Boletín de la Asociación española de Entomología. 1989. 13: 407-430.
28. Reina D., Breña M., Habela MA., Navarrete I. Parasitofauna de rumiantes menores en Cáceres. Mundo Ganadero. 1991. 7.
29. Reyes A., Molina C. Allozyme variation in a natural population of the horse fly *Haematopota italica* Meigen (Diptera, Tabanidae). Miscellánia Zoológica. 1997. 20. 1: 19-25.
30. Rodríguez NF., Tejedor-Junco MT., González-Martín M., Gutierrez C. *Stomoxys calcitrans* as possible vector of *Trypanosoma evansi* among camels in an affected area of the Canary Islands, Spain. Revista da Sociedade Brasileira de Medicina Tropical. 2014 Aug;47. 4:510–2.
31. ‌ Tamarit A., Gutierrez C., Arroyo R., Jimenez V., Zagalá G., Bosch I., Sirvent J., Alberola J., Alonso I., Caballero C . *Trypanosoma evansi* infection in mainland Spain. Veterinary Parasitology. 2010 Jan 20. 167. 1:74–6.
32. ‌Tolrá MC. Nuevos datos sobre los dípteros de España (Diptera: Scenopinidae, Xylomyidae, Lonchopteridae, Hippoboscidae, Nycteribiidae). Boletín de la Asociación española de Entomología. 1998. 22. 1-2: 75-79.
33. Tolrá MC. Nuevos datos sobre dípteros iberobaleares (Diptera: Orthorrhapha y Cyclorrhapha). Boletín de la Asociación española de Entomología. 2001. 25. 1-2: 53-95.
34. Tolrá MC. Dípteros nuevos para la Península Ibérica capturados en la provincia de Salamanca (España) (Insecta, Diptera). Boletín de la Sociedad Entomológica Aragonesa. 2004. 35: 187-194.
35. Tolrá MC. Algunos tábanos de Galicia y otras provincias españolas (España) (Diptera: Tabanidae). Arquivos Entomolóxicos. 2022. 25: 7-10.
36. Tolrá MC., Andersen H. Catálogo de los Diptera de España, Portugal y Andorra (Insecta). Monografías Sociedad Entomológica Aragonesa. 2002. 8. I.S.B.N.: 84 – 932807– 0 – 4.
37. Tolrá MC., Calvo F., Zabalegui I. Algunos dípteros capturados en el Parque Natural de Aiako Harria (País Vasco: Gipuzkoa) (Insecta: Diptera). Heteropterus Revista de Entomología. 2003. 3: 51-56.
38. Tolrá MC., Calvo F., Zabalegui I. Nuevos datos sobre dípteros capturados en el Parque Natural de Aiako Harria (España, País Vasco: Gipuzkoa) (Insecta: Diptera). Heteropterus Revista de Entomología. 2006. 6: 95-103.
39. Tolrá MC., Aguirre-Segura A. Algunos dípteros capturados en el Parque Natural Cabo de Gata-Níjar (Almería, España) (Insecta, Diptera). Boletín de la Sociedad Entomológica Aragonesa. 2007. 41: 197-202.
40. Tolrá MC., Portela JL., Pérez J., Pérez R. 212 especies de dípteros de Galicia (NO España) (Insecta: Diptera). Boletín BIGA. 2010. 9: 89-108.
41. Vallejo N., Aihartza J., Goiti U., Arrizabalga-Escudero A., Flaquer C., Puig X., Aldasoro M., Baroja U., Garin I. The diet of the notch-eared bat (*Myotis emarginatus*) across the Iberian Peninsula analysed by amplicon metabarcoding. Hystrix, the Italian Journal of Mammalogy. 2019. 30.
42. Vallejo N., Aihartza J., Olasagasti L., Aldasoro M., Goiti U., Garin I. Seasonal shift in the diet of the notched-eared bat (*Myotis emarginatus*) in the Basque Country: from flies to spiders. Mammalian Biology. 2023 Apr 12. 103. 4:419–31.
43. ‌Zeegers T., Batlle R., Almeida J. Dos nuevas especies de tabánidos (Diptera: Tabanidae) para la Península Ibérica. Boletín de la Sociedad Entomológica Aragonesa. 2012. 51: 149-152.
