# Supplementary material for: Geographical distribution of potential mechanical vectors implicated in Surra transmission in Spain: an entomological perspective
Source: Parasit Vectors. 2025 Jul 28;18:305. doi: 10.1186/s13071-025-06922-9 (PMC12305963; doi:10.1186/s13071-025-06922-9)
Supplement: Supplementary file 2 — Additional file 2. [file 13071_2025_6922_MOESM2_ESM.docx]

List of dipteran species of the Hippoboscidae, Muscidae and Tabanidae families cited in the consulted bibliography

| **Family** | **Genus** | **Species** | **Synonyms** | **Subespecies** | **Distribution** |
| --- | --- | --- | --- | --- | --- |
| **Hippoboscidae** | ***Hippobosca* Linnaeus, 1758** | *H. equina*  Linnaeus, 1758 | *H. equi*  Macquart,1835  *H. taurina*  *Rondani*, 1879 | No | General |
|  |  | *H. longipennis* Fabricius, 1805 | *H. capensis*  Olfers, 1816  *H. canina*  Drensky, 1926 | No | Mainland Spain |
|  | ***Lipoptena* Nitzsch, 1818** | *L. andaluciensis* González, 2024 |  | No | Mainland Spain |
|  |  | *L. cervi*  (Linnaeus, 1761) | *Melophagus trifasciata*  Olfers, 1816 *Ornithomyia nigrirostris*  Roser, 1840 | No | Mainland Spain |
|  |  | *L. couturieri*  Séguy, 1935 |  | No | Mainland Spain |
|  | ***Melophagus* Latreille, 1802** | *M. ovinus*  (Linnaeus, 1758) | *Hippobosca ovina* Linnaeus, 1758  *M. fera*  Speiser, 1908 | No | General |
| **Muscidae** | ***Haematobia* Lepeletier & Serville, 1828** | *H. Irritans*  (Linnaeus, 1758) | *Lyperosia meridionalis*  Bezzi, 1911  *Stomoxys pungens* Fabricius, 1787 | No | Mainland Spain |
|  |  | *H. titilans*  (Bezzi, 1907) | *Lyperosia titilans* Bezzi, 1907 | No | Canary Islands |

|  | ***Stomoxys* Geoffroy, 1762** | *S. calcitrans*  (Linnaeus, 1758) | *Conops calcitrans* Linnaeus, 1758  *Musca calcitrans* Harris, 1780 | No | General |
| --- | --- | --- | --- | --- | --- |
| **Tabanidae** | ***Atylotus***  **Osten-**  **Sacken, 1876** | *A. agrestis*  (Wiedemann, 1828) | *Tabanus agrestis*  Wiedemann, 1828 | No | Mainland Spain |
|  |  | *A. ffavoguttatus*  (Szilády, 1915) | *Ochrops ffavoguttatus*  Szilády, 1915 | No | Mainland Spain |
|  |  | *A. fulvus*  (Meigen, 1804) | *A.*  *aurisquamatus*  Bigot, 1892 | Yes | Mainland Spain |
|  |  | *A. latistriatus*  Brauer, 1880 | *A. nigrifacies*  Gobert, 1880 | No | Mainland Spain Balearic Islands |
|  |  | *A. loewianus*  (Villeneuve, 1920) | *Orchops loewianus* Villeneuve, 1920 | No | Mainland Spain Balearic Islands |
|  |  | *A. mallorcanus*  (Enderlein, 1925) | *Dasystypia mallorcana* Enderlein, 1925 | No | Balearic Islands |
|  |  | *A. quadrifarius*  (Loew, 1874) | *A.*  *afghanistanicus* Moucha C Chvála, 1959 | No | Mainland Spain |
|  |  | *A. rusticus*  (Linnaeus, 1767) | *A.*  *parallelliformis*  (Szilády, 1923) | No | Mainland Spain |
|  |  | *A. venturii*  Leclerq, 1967 |  | No | Mainland Spain |
|  | ***Chrysops***  **Meigen, 1803** | *C. caecutiens*  (Linnaeus, 1758) | *C. clarus*  Goffe, 1931 | Yes | Mainland Spain |

|  |  | *C. connexus*  Loew, 1858 | *C. aurantiacus*  Jaennicke, 1866 | No | Mainland Spain |
| --- | --- | --- | --- | --- | --- |
|  |  | *C. flavipes*  Meigen, 1804 | *C. abdominalis*  Kröber, 1920 | Yes | Mainland Spain |
|  |  | *C. italicus*  Meigen, 1804 | *C. fenestrarus*  (Fabricius, 1794) | No | Mainland Spain |
|  |  | *C. mauritanicus*  Costa, 1893 | *C. chobauti*  Villeneuve, 1934 | No | Mainland Spain |
|  |  | *C. relictus*  Meigen, 1820 | *C. chlorosis*  Goffe, 1931 | No | Mainland Spain |
|  |  | *C. viduatus*  (Fabricius, 1794) | *C. intermedius*  Goffe, 1931 | No | Mainland Spain |
|  | ***Dasyrhamphis* Enderlein, 1922** | *D. atra*  (Rossi, 1790) | *D. ater*  (Rossi, 1790) | No | Mainland Spain |
|  |  | *D. denticornis*  (Enderlein, 1925) | *Sziladya denticornis* Enderlein, 1925 | No | Mainland Spain |
|  |  | *D. nigrita*  (Fabricius, 1794) | *Sziladya apicalis*  Enderlein, 1925 | No | Mainland Spain |
|  | ***Haematopota***  **Meigen, 1803** | *H. bigoti*  (Gobert, 1880) | *H.*  *monspellensis* Villeneuve, 1921 | Yes | Mainland Spain |
|  |  | *H. crassicornis*  Wahlberg, 1848 | *Chrysozona ffavopilosa* Kröber, 1922 | No | Mainland Spain |
|  |  | *H. csikii*  Szilády, 1922 | *H. perisi*  Salom, 1961 | No | Mainland Spain |
|  |  | *H. enriquei*  Leclerq, 1971 |  | No | Mainland Spain |

|  |  | *H. eugeniae*  Portillo y Schacht, 1982 |  | No | Mainland Spain |
| --- | --- | --- | --- | --- | --- |
|  |  | *H. grandis*  Meigen, 1820 | *Chrysozona grandis* (Meigen, 1820) | No | Mainland Spain |
|  |  | *H. italica*  Meigen, 1804 | *H. argyrophora*  Kröber, 1922 | Yes | Mainland Spain |
|  |  | *H. lambi*  Villeneuve, 1921 | *Chrysozona planicornis* Kröber, 1922 | No | Mainland Spain |
|  |  | *H. ocelligera*  (Kröber, 1922) | *H. hispanica*  Szilády, 1923 | No | Mainland Spain |
|  |  | *H. pandazisi*  (Kröber, 1936) |  | No | Mainland Spain |
|  |  | *H. pluvialis*  (Linnaeus, 1758) | *Tabanus pluvialis* Linnaeus, 1758 | Yes | Mainland Spain |
|  |  | *H.*  *pseudolusitanica*  Szilády, 1923 |  | No | Mainland Spain |
|  | ***Hybomitra* Enderlein, 1922** | *H. aterrima*  (Meigen, 1820) | *Tabanus aterrimus* Meigen, 1820 | No | Mainland Spain |
|  |  | *H. caucasica*  (Enderlein, 1925) | *H. olsoufievina*  Philip, 1961 | No | Mainland Spain |
|  |  | *H. ciureai*  (Séguy, 1937) |  | No | Mainland Spain |
|  |  | *H. distinguenda*  (Verrall, 1909) | *Tabanus distinguendus* Verrall, 1909 | No | Mainland Spain |
|  |  | *H. expollicata*  (Pandellé, 1883) | *Tabanus expollicatus* Pandellé, 1883 | Yes | Mainland Spain Balearic Islands |

|  |  | *H. micans* (Meigen, 1804) | *Tabanus micans*  Meigen, 1804 | No | Mainland Spain |
| --- | --- | --- | --- | --- | --- |
|  |  | *H. montana*  (Meigen, 1820) | *Tabanus montana*  Meigen, 1820 | Yes | Mainland Spain |
|  |  | *H. solstitialis*  (Meigen, 1820) | *Tabanus solstitialis* Meigen, 1820 | No | Mainland Spain |
|  |  | *H. tumojosoi*  Schacht & Portillo, 1982 | *H. alegrei*  Travassos, 1984 | No | Mainland Spain |
|  |  | *H. valenciae*  (Leclerq, 1957) | *Therioplectes valenciae*  Leclerq, 1957 | No | Mainland Spain |
|  |  | *H. vittata*  (Fabricius, 1794) | *Tabanus vittatus* Fabricius, 1794 | No | Mainland Spain |
|  |  | *H. zaballosi*  Portillo, 1988 |  | No | Mainland Spain |
|  | ***Nemorius* Rondani, 1856** | *N. vitripennis*  (Meigen, 1820) | *Chrysops kondarensis* Baratov, 1961 | No | Mainland Spain |
|  | ***Pangonius* Latreille, 1802** | *P.*  *(Melanopangonius) brevicornis* (Kröber, 1921) |  | No | Mainland Spain |
|  |  | *P.*  *(Melanopangonius) haustellatus*  (Fabricius, 1781) | *Bombylius haustellatus* Olivier, 1789 | No | Mainland Spain |
|  |  | *P.*  *(Melanopangonius) micans*  Meigen, 1820 |  | No | Mainland Spain |
|  |  | *P. affinis* (Loew,  1859) |  | No | Mainland Spain |

|  |  | *P. dimidiatus*  (Loew, 1859) |  | No | Mainland Spain |
| --- | --- | --- | --- | --- | --- |
|  |  | *P. escalerae*  Strobl, 1906 |  | No | Mainland Spain |
|  |  | *P. ferrugineus*  (Meigen, 1804) | *Tanyglossa ferruginea* Meigen, 1804 | No | Mainland Spain |
|  |  | *P. fumidus* (Loew, 1859) | *Pangonia loewii*  Kröber, 1921 | No | Mainland Spain |
|  |  | *P. granatensis*  (Strobl, 1906) |  | No | Mainland Spain |
|  |  | *P. griseipennis*  (Loew, 1859) |  | No | Mainland Spain |
|  |  | *P. hermanni*  (Kröber, 1921) |  | No | Mainland Spain |
|  |  | *P. obscuratus*  (Loew, 1859) |  | No | Mainland Spain |
|  |  | *P. sobradieli*  (Séguy, 1934) |  | No | Mainland Spain |
|  |  | *P. variegatus*  (Fabricius, 1805) | *Pangonia acutipalpis*  Kröber, 1921 | No | Mainland Spain |
|  | ***Philipomyia* Olsufjev, 1964** | *P. aprica*  (Meigen, 1820) | *Tabanus apricus*  Meigen, 1820 | No | Mainland Spain |
|  |  | *P. graeca*  (Fabricius, 1794) | *Tabanus ferrugineus*  Meigen, 1804 | No | Mainland Spain |
|  | ***Silvius***  **Meigen, 1820** | *S. alpinus*  (Scopoli, 1763) | *Tabanus vituli* Fabricius,1805 | No | Mainland Spain |
|  |  | *S. appendiculatus*  Macquart, 1846 |  | No | Mainland Spain |
|  |  | *S. variegatus*  (Fabricius, 1805) | *Chrysops singularis*  Meigen, 1835 | No | Mainland Spain |

|  | ***Tabanus* Linnaeus, 1758** | *T. albifrons*  Szilády, 1914 | *Dasystypia schmidtiana* Enderlein, 1931 | No | Mainland Spain |
| --- | --- | --- | --- | --- | --- |
|  |  | *T. autumnalis*  Linnaeus, 1761 | *T. anthracinus*  Walker, 1851 | Yes | Mainland Spain Balearic Islands |
|  |  | *T. barbarus*  Coquebert, 1804 | *T. auricinctus*  Macquart, 1838 | No | Mainland Spain |
|  |  | *T. bifarius*  Loew, 1858 | *Dasystipia mallorcana* Enderlein, 1925 | No | Mainland Spain |
|  |  | *T. bovinus*  Linnaeus, 1758 | *T. auratus*  Ghidini, 1935 | No | Mainland Spain |
|  |  | *T. brassofortei*  Dias, 1980 |  | No | Mainland Spain |
|  |  | *T. briani*  Leclerq, 1962 | *T. carpathicus*  Chvála, 1964 | No | Mainland Spain |
|  |  | *T. bromius*  Linnaeus, 1758 | *Straba simplex* Muschamp, 1939 | Yes | Mainland Spain Balearic Islands |
|  |  | *T. castellanus*  Strobl, 1906 |  | No | Mainland Spain |
|  |  | *T. cognatus*  Loew, 1858 |  | No | Mainland Spain |
|  |  | *T. cordiger*  Meigen &  Wiedemann, 1820 | *T. fortunatus*  Frey, 1936  *T. braueri*  Jaennicke, 1866 | No | Mainland Spain Balearic Islands Canary Islands |
|  |  | *T. darimonti*  Leclerq, 1964 |  | No | Mainland Spain |
|  |  | *T. eggeri*  Schiner, 1868 | *T. intermedius*  Egger, 1859 | No | Mainland Spain |

|  |  | *T. exclusus*  Pandellé, 1883 |  | No | Mainland Spain |
| --- | --- | --- | --- | --- | --- |
|  |  | *T. ffavofemoratus*  Strobl, 1909 |  | No | Mainland Spain Balearic Islands |
|  |  | *T. glaucopis*  Meigen, 1820 | *T. lunulatus*  Meigen, 1820 | Yes | Mainland Spain Balearic Islands |
|  |  | *T. indrae*  Hauser, 1939 | *T. montivagus*  Olsufiev, 1970 | No | Mainland Spain |
|  |  | *T. lunatus*  Fabricius, 1794 | *T. algiricus*  Thunberg, 1827 | No | Mainland Spain |
|  |  | *T. maculicornis*  Zetterstedt, 1842 | *T. glaucus*  Walker, 1851 | No | Mainland Spain |
|  |  | *T. martini*  Kröber, 1928 |  | No | Mainland Spain |
|  |  | *T. miki*  Brauer, 1880 | *T. australis*  Hauser, 1960 | No | Mainland Spain |
|  |  | *T. nemoralis*  Meigen, 1820 | *T. batnensis*  Bigot, 1892 | No | Mainland Spain |
|  |  | *T. paradoxus*  Jaennicke, 1866 | *T. macedonicus*  Kröber, 1936 | No | Mainland Spain |
|  |  | *T. quatournotatus*  Meigen, 1820 |  | No | Mainland Spain |
|  |  | *T. rectus*  Loew, 1858 | *T. ornatus*  Jaennicke,1866 | No | Mainland Spain |
|  |  | *T. regularis*  Jaennicke, 1866 |  | No | Mainland Spain |
|  |  | *T. rupium*  (Brauer, 1880) | *Atylotus rupium*  Brauer, 1880 | No | Mainland Spain |

|  |  | *T. spectabilis*  Loew, 1858 | *T. aspahanicus*  Rondani, 1873 | No | Mainland Spain |
| --- | --- | --- | --- | --- | --- |
|  |  | *T. spodopterus*  Wiedemann, 1820 | *T. ibericus* Olsufiev, Moucha &  Chvála, 1967 | No | Mainland Spain |
|  |  | *T. spodopteroides* Olsufiev, Moucha & Chvála, 1969 |  | No | Mainland Spain |
|  |  | *T. sudeticus*  Zeller, 1842 | *T. confusus*  Goffe, 1931 | No | Mainland Spain |
|  |  | *T. tergestinus*  Egger, 1859 |  | No | Mainland Spain Balearic Islands |
|  |  | *T. tinctus*  Walker, 1850 | *T. mixtus*  Szilády, 1914 | No | Mainland Spain |
